# Flexural Strengthening of Stone Masonry Walls Using Textile-Reinforced Sarooj Mortar

**DOI:** 10.3390/ma16165703

**Published:** 2023-08-20

**Authors:** Abdullah Hilal Al-Saidy, Manal Al-Busaidi, Sherif El-Gamal, Kazi Md Abu Sohel

**Affiliations:** 1 Department of Civil and Architectural Engineering, Sultan Qaboos University, Muscat 123, Oman; sherif@squ.edu.om (S.E.-G.); kmasohel@squ.edu.om (K.M.A.S.); 2 Petrofac E & C, Muscat 111, Oman

**Keywords:** strengthening, historical, stone masonry, TRM, textile

## Abstract

The majority of historical buildings and structures in Oman were built using unreinforced stone masonry. These structures have deteriorated due to ageing of materials, environmental degradation, and lack of maintenance. This research investigates the physical, chemical, and mechanical properties of local building materials and the results of an experimental study on the out-of-plane bending effectiveness of an innovative strengthening method applied to existing masonry walls. The technique consists of the application of a basalt textile-reinforced sarooj mortar (TRM) on one face of the walls. Bending tests of masonry wall samples (1000 mm width, 2000 mm height, and 350 mm depth) were carried out on one unreinforced specimen and three different cases of reinforced specimens. The performance of unreinforced and reinforced specimens was analyzed and compared. The strengthened specimens were able to resist moments of out-of-plane bending 2.5 to 3 times greater than those of unreinforced specimen (160–233% increase). Moreover, the strengthened walls were able to sustain higher deformations (deflections) than the unreinforced specimen ranging from 20 to 130%. The results showed that using TRM was effective for the out-of-plane strengthening of stone masonry using a local material (sarooj) that is compatible with existing stone masonry building materials.

## 1. Introduction

Stone masonry is a traditional construction technique that is practiced wherever stones are locally available [1]. Oman has over 500 forts, castles, and towers [2]. Locally available materials were used in the construction of historical structures, since local materials were cheap and compatible with the surrounding environment. Oman is known for its variation in topography, with the different materials available used in construction.

In historical buildings, the main structural elements are unreinforced masonry (URM) walls. These elements mainly carry compressive vertical gravity loads and are too weak to resist seismic activity or any considerable lateral loads [3]. Therefore, it is necessary to strengthen unreinforced walls against flexural and shear loads. Stones or mud bricks were typically used in the construction of URM walls. A special mortar was used to join the stones and mud bricks. This special mortar consisted of sarooj, lime, and water. Sarooj is a local term for calcined clay used as artificial pozzolan. Sarooj mixed with lime was used in all historical structures prior to the availability of Portland cement [4].

Various conventional techniques have been used for the retrofitting of masonry walls, such as grout injection, the use of shotcrete jackets, or the use of concrete skin [5,6]. These techniques add additional weight to the building, which increases the threat of failure under cyclic loading. Additionally, the reinforcement may corrode in the long term and this traditional technique requires heavy equipment and scaffolding. These techniques also change the appearance of the retrofitted structure, causing alterations in the original historical value of the building [5,6].

Fiber-reinforced polymers (FRPs) have become increasingly popular for strengthening structures due to their favorable properties, including their high strength-to-weight ratio, ease of application, and corrosion resistance and the minimal change to the geometry of the strengthened structure. Despite these advantages, the FRP strengthening technique has a few drawbacks, which are attributed to the organic resins used to bind the fibers. These disadvantages may be summarized as follows: (1) thermal incompatibility; (2) the relatively high cost of epoxy resins; (3) difficulty to apply on wet surfaces; and (4) lack of vapor permeability [7,8,9,10,11,12,13].

To overcome these drawbacks, cement-based mortar can be used instead of epoxy resins and grid textiles can be used instead of continuous fiber sheets. Combining a mortar matrix with textile reinforcement is known as textile-reinforced mortar (TRM). Various textile fibers are available including glass, carbon, basalt, aramid, polypropylene (PP), polyparaphenyle benzodioxol (PBO), or steel.

TRM is used as a strengthening/retrofitting material for existing structures or for the construction of new structures. The application of TRM as a strengthening and retrofitting material for URM structures is based on its high strength-to-weight ratio and its chemical and mechanical compatibility with masonry substrates [7].

Relatively limited research has been performed on the retrofitting and strengthening of stone masonry structures [12,13,14,15,16,17,18]. Research has, however, been conducted on ordinary concrete block masonry walls, sand lime bricks, and rubble stone walls. Strengthening research covers static and dynamic responses; single or double wythe walls; textile covering one or both sides of the wall; the number of textile layers used in the strengthening scheme; and the type of textile fiber. Almost all studies reported that using TRM in the strengthening of walls enhanced the out-of-plane bending capacity compared with URM walls. As mentioned earlier, Oman has an inventory of 500 castles and forts, all of which were built with unreinforced masonry; further investigation is needed especially in the case of historical structures.

Local materials should be used as much as possible to preserve Oman’s historical buildings. A study by Hago and Al-Rawas [19] reported that the sarooj properties depend on the clay mineral components, such as silica and alumina. Reactive silica and alumina’s reaction with lime and water provide the binding nature of sarooj in the form of calcium silicate and aluminates hydrates, like those found in Portland cement. The chemical composition, the physical properties of sarooj, and the burning temperature affect sarooj reactivity [20]. Another study also recommended a cement/sarooj ratio of 0.6 and a sand/sarooj ratio of 0.3 to achieve the highest compressive strength for all mixes [21].

The presented experimental work in this article evaluates the efficiency of using TRM to strengthen stone masonry walls through constructing and strengthening the representative wall specimens. The study starts by conducting a chemical analysis and physical tests on limestone and sarooj to determine their various properties. Then, masonry walls were constructed using the selected materials. Strengthening schemes were applied to the wall specimens using basalt fiber textiles bonded with mortar made of sarooj.

## 2. Material Properties

### 2.1. Limestone

**Figure 1:**
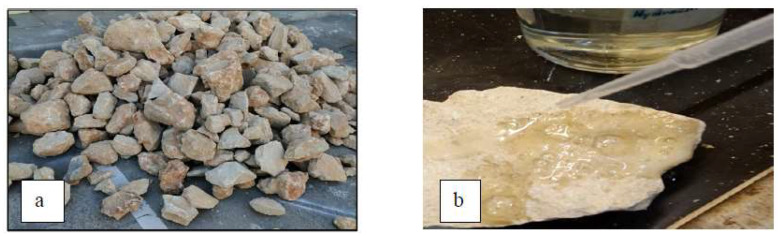
(**a**) Limestone, (**b**) limestone reaction with HCl.

**Figure 2:**
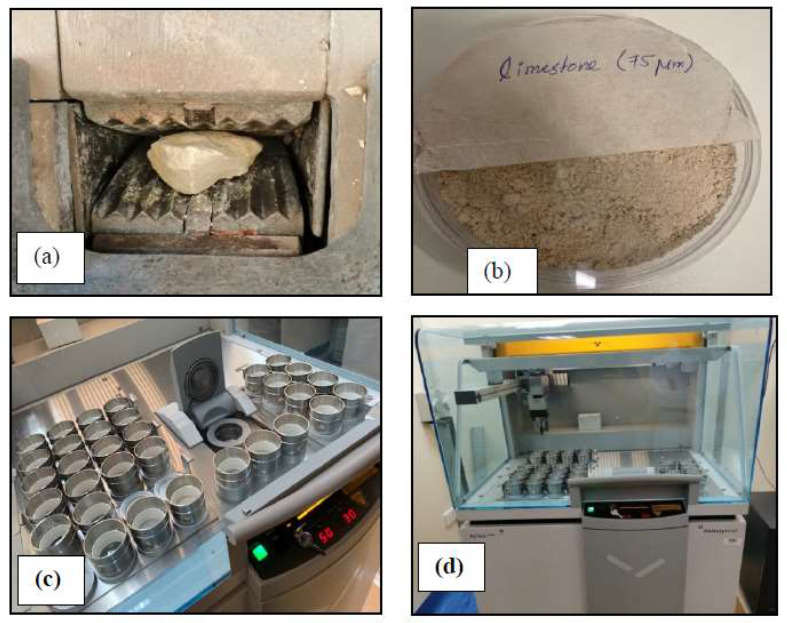
Photographs of (**a**) crushing stone sample; (**b**) sieved sample; (**c**,**d**) XRF Test.

Limestone was widely used in the construction of Oman’s historical buildings due to its availability, ease of cutting, and aesthetic appearance. It is defined according to ASTM C51, 2011 [22] as sedimentary rock consisting chiefly of calcium carbonate or calcium and magnesium carbonates. The stone used in this study was limestone collected from a nearby source (see Figure 1a). Hydrochloric acid was used to initially verify the limestone by the reaction of hydrochloric acid (HCl) with calcium carbonate (CaCo) to produce a fizzing reaction as CO_2_ is released from the reaction of HCl with CaCO_3_ (see Figure 1b). A standard procedure was followed to determine the stone’s chemical composition [23]. The procedure requires a crushed sample of the stone in the form of particles, with a passing sieve size of 0.075 mm, is prepared for the chemical analysis (see Figure 2). The chemical analysis results are summarized in Table 1. The major element of the stone was found to be CaO (68%), which indicates that the stone is limestone.

**Figure 3:**
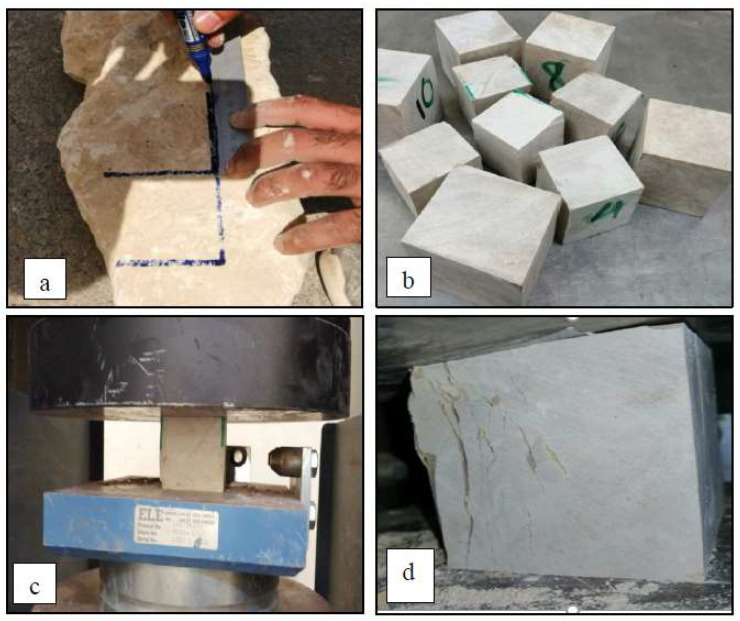
Photographs of (**a**,**b**) cutting and extracting stone cube samples; (**c**,**d**) compressive test and failure modes.

The uniaxial compressive strength of the limestone was determined by extracting 10 cubes with dimensions of approximately 70.7 × 70.7 × 70.7 mm from random limestone samples (see Figure 3). The uniaxial compressive strength was determined as per ASTM D7012, 2004 [24]. It was found to be 57 MPa, as an average value from 10 samples (with a standard deviation of 8 MPa).

The water absorption of the limestone was determined according to ASTM D6473, 2010 [25] standard procedures. The limestones were soaked in water; they were then oven dried and weighed. The water absorption of limestone was determined to be 2.2%.

### 2.2. Sarooj

Sarooj was used in the construction of historical buildings for centuries. Previous studies [4,19,20,21] have shown that the reactivity of sarooj is influenced by different factors, including its physical, mechanical, chemical, and geotechnical properties.

The sarooj used in this study was donated by the Ministry of Heritage and Culture (MHC). The MHC maintenance department uses sarooj extensively in the restoration work of historical buildings. A chemical analysis of sarooj was conducted using a sample of sarooj with a passing sieve of 0.075 mm according to standard procedures to determine its chemical composition [26]. The results of the chemical analysis are summarized in Table 1. Three major components are considered to define sarooj as a pozzolanic material: SiO_2_, Al_2_O_3_, and Fe_2_O_3_. The total of the three components should exceed 70% according to ASTM Standard C618 [27]. However, in this sample, the total of the three components was approximately 52%. Therefore, the sarooj obtained from the MHC cannot be considered as a natural pozzolana and may need the addition of some cement. The source of the clay used to produce sarooj and the calcination process affects the chemical composition of sarooj. In the traditional way of producing sarooj, it is difficult to control the calcination process [4].

### 2.3. Bed Joints Mortar

**Figure 4:**
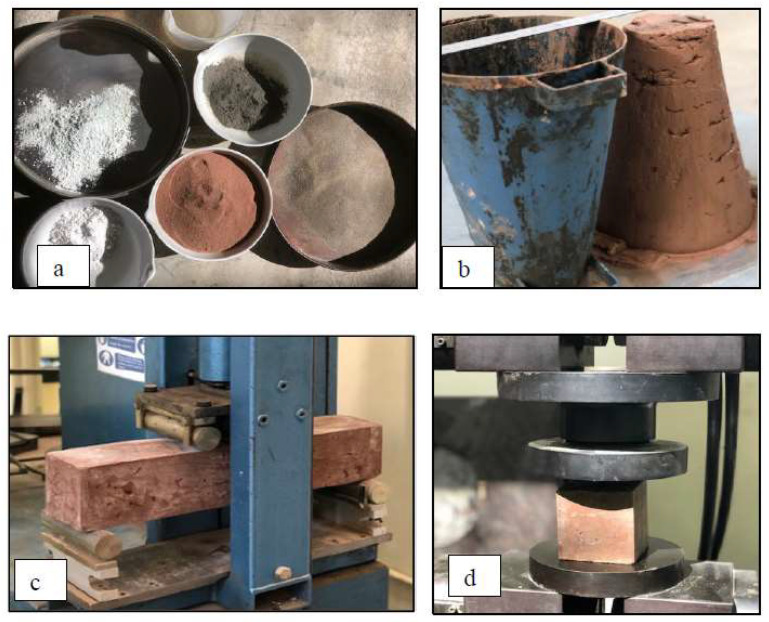
Jointing mortar (**a**) materials used; (**b**) slump test; (**c**) flexural test; (**d**) compression test.

The cementing materials used in the mixtures were sarooj (passed a 600 µm sieve), ordinary Portland cement (OPC), white cement, and lime. Cement was added to enhance the binding properties of the mortar as recommended by Hago et al. [21]. White and gray cement were used in the jointing mortar, while only white cement was used in the plaster (matrix) mortar to preserve the natural color of sarooj. Fine sand (passed through a 600 µm sieve) was used in all mixtures to avoid shrinkage and micro-cracking. The matrix of the jointing mortar mix was sarooj, sand, ordinary Portland cement, white cement, lime, and water with proportions by weight of 1:0.4:0.2:0.2:0.1:0.5, respectively (see Figure 4). This mix was selected from several mix trials based on workability and mechanical properties. Each mixture was cast into metal molds, producing three identical cubes with dimensions of 70.7 × 70.7 × 70.7 mm and prisms with dimensions of 100 × 100 × 500 mm. All mixtures were cured at a lab temperature of 22 ± 2 °C and a relative humidity of 20–30%. The slump for the mix was measured to be 35 mm. The presence of sarooj is the reason for the low slump; sarooj absorbs more water compared to sand. A uniaxial compressive test of the cubes was conducted according to ASTM D7012, 2004 [24] and resulted in an average 28-day compressive strength of 11.39 MPa.

### 2.4. Plaster Mortar

The plaster mortar mix was sarooj, sand, white cement, lime, and water with proportions by weight of 1:0.5:0.5:0.375:0.75, respectively (see Figure 4). The measured slump for the plaster mortar mix was measured to be 34 mm. This mix was selected from several mix trials based on workability and mechanical properties. A similar procedure as used in the jointing mortar was adopted in casting and curing the specimens. The recommendations by Hago et al. [21] were adopted for the selected optimum design mix ratios of sarooj, sand, and cement to produce the maximum strength. The uniaxial test of the cubes resulted in an average 28-day compressive strength and tensile strength of 11.67 MPa and 1.7 MPa, respectively.

### 2.5. Basalt Textile

**Figure 5:**
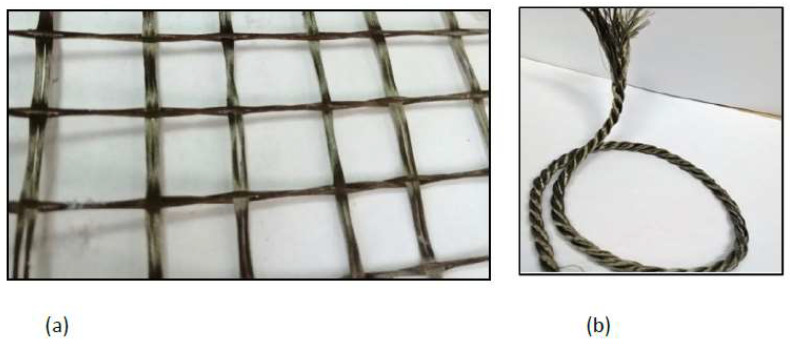
(**a**) Basalt fiber textile, (**b**) basalt rope.

A basalt textile was used in the strengthening schemes of the wall specimens. The textile consisted of a bi-directional grid that has a roving dimension of 1.6 mm wide by 0.5 mm thick in the longitudinal direction and a roving dimension of 0.6 mm wide by 0.5 mm thick in the transverse direction. The spacing between the rovings is 10 mm in both directions (Figure 5a).

**Figure 6:**
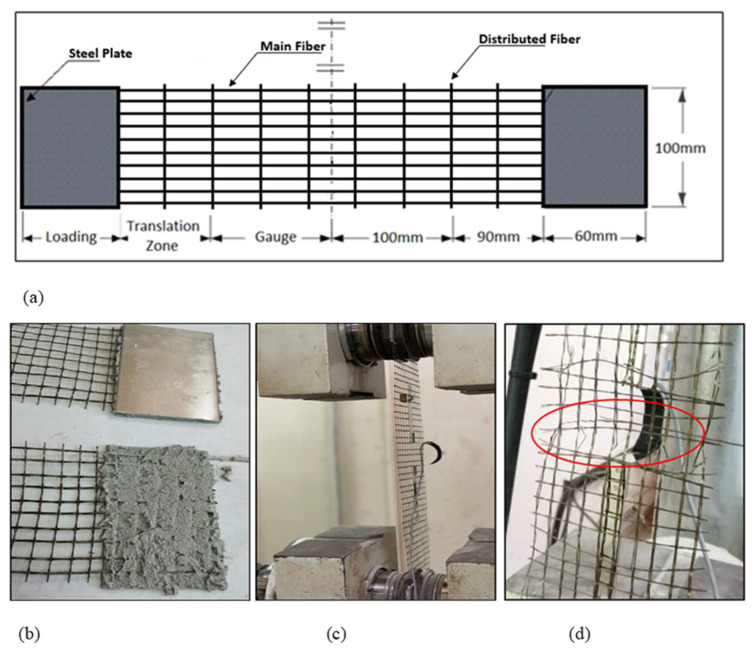
Tensile test set up: (**a**) sketch of basalt textile grid testing sample; (**b**) preparing the sample with steel plate ends; (**c**,**d**) test set up with LVDT.

**Figure 7:**
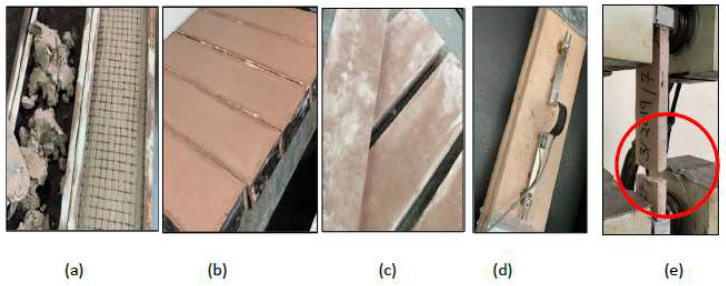
Composite samples (basalt + mortar) tensile test set up: (**a**–**c**) preparing the sample with steel plate ends; (**d**) test set up with LVDT; (**e**) failure mode.

**Figure 8:**
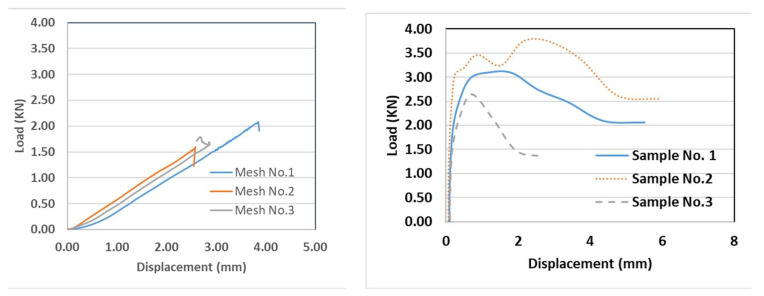
Tensile load vs. elongation of tested samples.

A uniaxial tensile strength was conducted according to ASTM D5034—09(2013) [28]. The textile tensile test samples were cut to be 500 mm long and 100 mm wide, as shown in Figure 6. In addition, composite coupon specimens consisting of one layer of textile fibers embedded in sarooj mortar were also tested to determine the uniaxial tensile strength as shown in Figure 7. First, a 500 mm long, 100 mm wide, and 22 mm thick panel was cast on a flat wooden mold. Once one layer of mortar was cast and smoothened, one ply of the textile grid was applied. Then, the cover layer of mortar was cast to fill the mold as shown in Figure 7. The composite specimens were cured at room temperature for 28 days. A uniaxial load was monotonically applied in a displacement-controlled routine. Metal plates of approximately 2.5 mm thickness were attached to the ends of test specimens using grout to avoid stress concentration beneath the clamp. Figure 8 shows the tensile test results of the textile and the composite specimens. The load versus the displacement of the textile fiber showed a linear curve until failure, while the curve for the composite shows two characteristic phases: an initial steep curve representing the non-cracked section phase and then a reduced slope curve corresponding to the cracked section phase. The failure of the textile specimens was characterized by the successive rupture of the weft yarns. The rupture occurred randomly, as shown in Figure 6d. The failure of the composite specimens was due to the complete rupture of the specimen, as shown in Figure 7e. The peak load for the fiber mesh reached an average of 1.7 kN, while the peak load for the composite specimens reached an average of 3.0 kN. The mortar matrix in the composite specimens helped to distribute the load among the textile fibers, resulting in a higher load resistance.

## 3. Stone Masonry Wall Construction and Test Setup

### 3.1. Compressive Strength of Masonry

**Figure 9:**
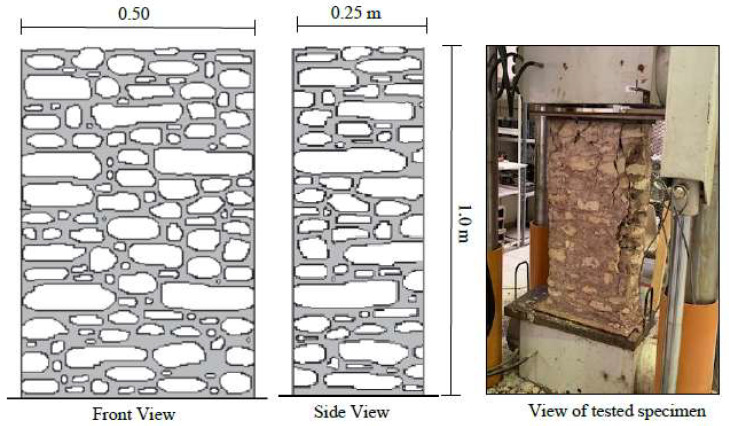
Schematic and photographs of the stone masonry wall specimens.

**Figure 10:**
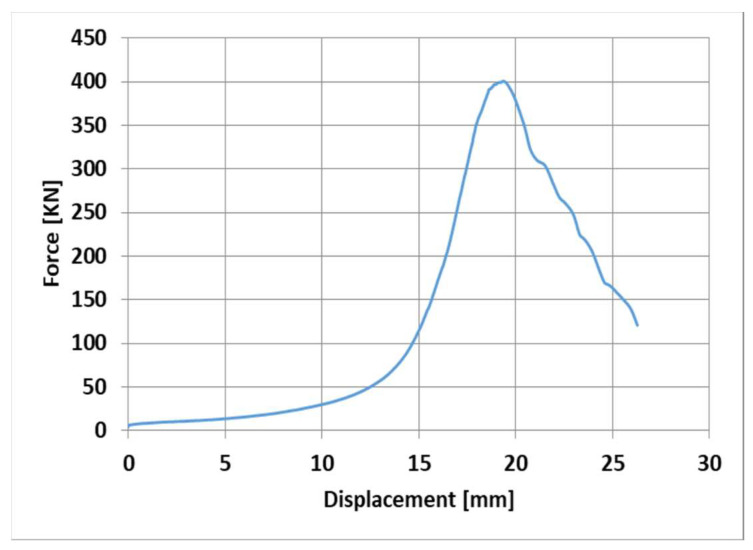
Load vs. displacement of the stone masonry wall specimens.

Two specimens were constructed with dimensions of 500 mm long, 250 mm wide, and 1000 mm high, as sketched in Figure 9. They were unreinforced walls consisting of only limestone and jointing mortar. These walls were constructed to evaluate the compressive strength of the stone masonry. The wall specimens were tested under compression (monotonic load) using a 4000 kN universal testing machine, as illustrated in Figure 9. The load was applied by a hydraulic cylinder, which was computer controlled at a rate of 0.6 kN/s, and was measured using the machine’s load cell. Displacement was measured via a displacement transducer. A typical load displacement curve is shown in Figure 10. Initially, the curve looks flat, as the load increases after some displacement. This is due to the neoprene (rubber) pad at the top of the wall at the point of the load application and due to the squeezing of the mortar layers. The stones then pick up the load resistance and the load–displacement curve turns into a steep curve, as illustrated in Figure 10. The load then drops once the peak load is reached. The wall suffered from vertical cracks due to the effect of Poisson’s ratio (see Figure 9). The wall then started to crumble, at which point the test was stopped. The average compressive strength from the two walls specimens was 3.0 MPa.

### 3.2. Wall Specimens

**Figure 11:**
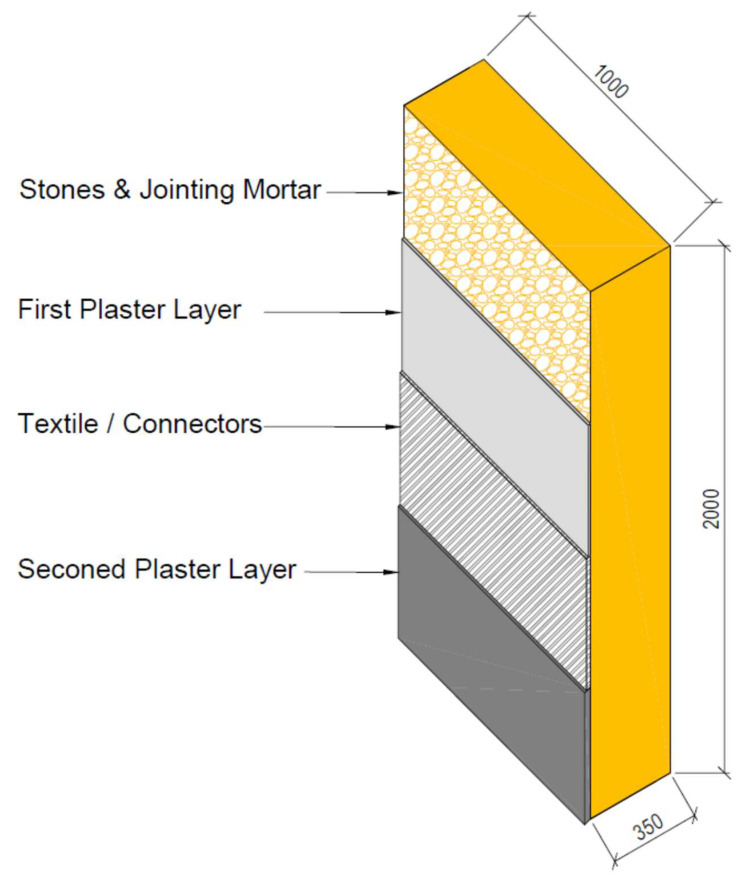
Schematic illustration (units in mm) of strengthened wall specimens.

The experimental program of supporting the stone wall specimens consisted of strengthening 2000 mm high, 1000 mm wide, and 350 mm thick limestone walls with basalt TRM. This type of masonry is common in the masonry buildings of a large part of Oman and other countries. Four different walls were constructed to be tested for bending. One specimen was not reinforced (URM) and the other three specimens were reinforced with basalt textile mesh. The details of each wall are illustrated below, and a schematic illustration of the geometry of the walls is shown in Figure 11.

The samples were defined using an identifier composed of three parts. The first part indicates whether is was unreinforced masonry (U) or a strengthened wall with basalt mesh (R), and the latter letters identify the type of reinforcement (C = control wall without textile, T = wall with textile, TS = wall with textile and screws, and TR = wall with textile and basalt ropes). A description of the wall specimens is listed in Table 2.

#### 3.2.1. CASE I: Unreinforced Masonry Wall (UC)

**Figure 12:**
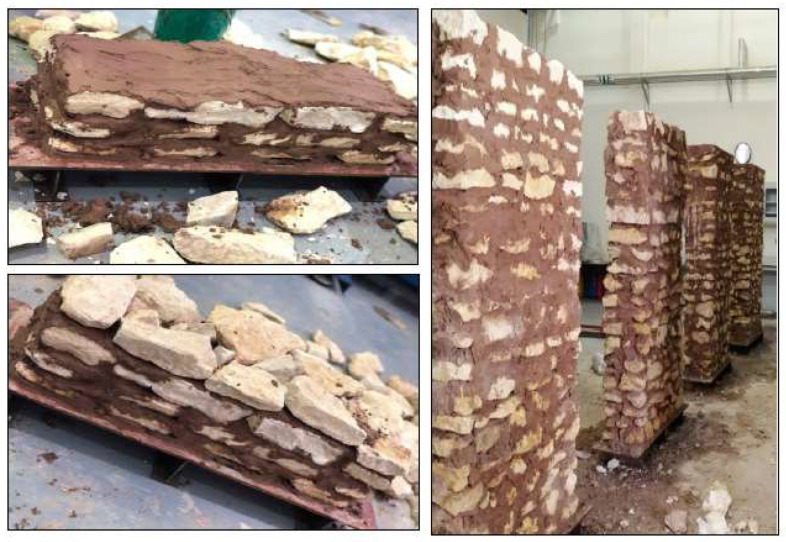
Construction of stone masonry wall specimens.

This wall is considered the reference wall. It was constructed from stones and jointing mortar only. All walls were constructed by layering alternative layers of stone and mortar. Each wall was constructed until reaching a height of approximately 60 cm and the wall was then left for the mortar to set (24 h). The construction of the following layers was then resumed until reaching a full height of 2.0 m. Figure 12 shows photos of the construction of the unreinforced masonry wall specimens.

#### 3.2.2. CASE II: Wall Reinforced by Basalt Textile (RT)

**Figure 13:**
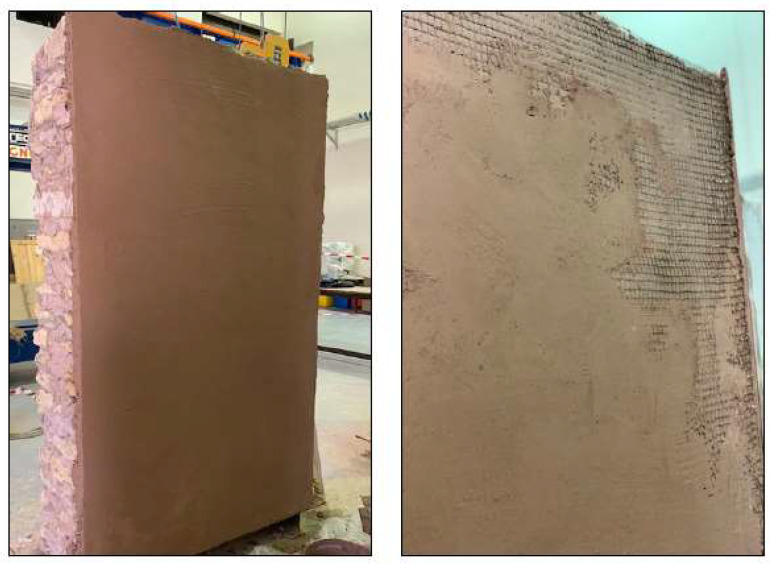
Strengthening of first wall with one textile layer (RT).

The second wall was strengthened using basalt fiber textile. The first layer of sarooj mortar was applied to one face of the wall (tension face) to receive the textile mesh. The first layer of plaster varied between 2 and 3 cm to level the face to stick the basalt mesh. A textile layer then was applied and pressed by hand to stick to the first layer of mortar. A second plaster layer of 0.5 cm thick was applied to cover the mesh. Photos of the wall construction are presented in Figure 13.

#### 3.2.3. CASE III: Wall Reinforced by Basalt Textile and Stainless-Steel Screws and Washers (RTS)

**Figure 14:**
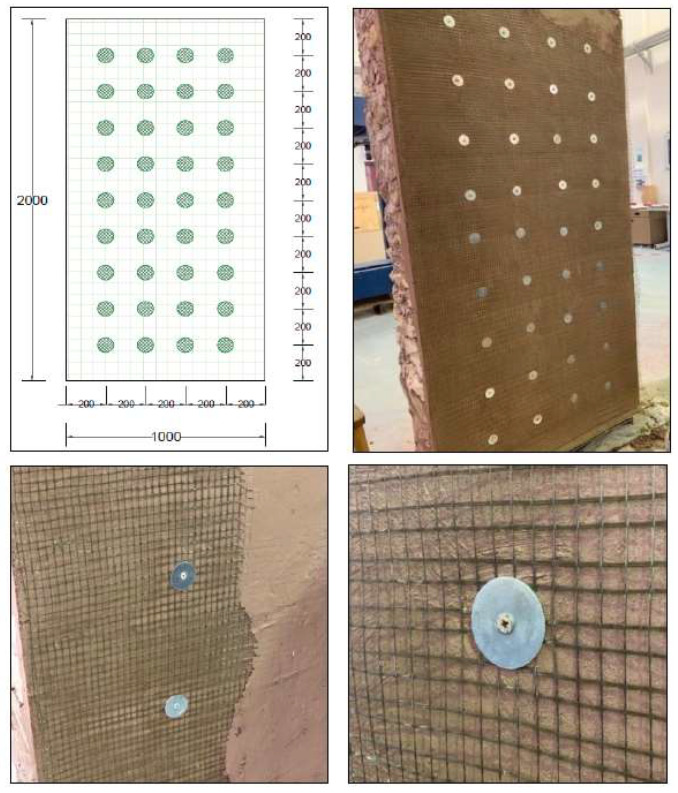
Strengthening of the second wall with one textile layer + screws (RTS).

The third wall was strengthened using a basalt mesh and stainless steel screws and washers. Holes for the screws were first drilled into the walls. The distance between the holes was 20 cm in both directions, as shown in Figure 14. Brass screws with plastic plugs were inserted inside the holes. A layer of sarooj mortar was then applied in preparation for the textile mesh. A textile layer was then applied and pressed by hand to stick to the first layer of mortar. After applying the basalt mesh, the brass screws were removed and reinserted again with steel washers to connect the mesh to the wall, as illustrated in Figure 14. A second plaster layer of 0.5 cm thick was applied to cover the mesh.

#### 3.2.4. CASE IV: Wall Reinforced by Basalt Mesh and Basalt Ropes (RTR)

**Figure 15:**
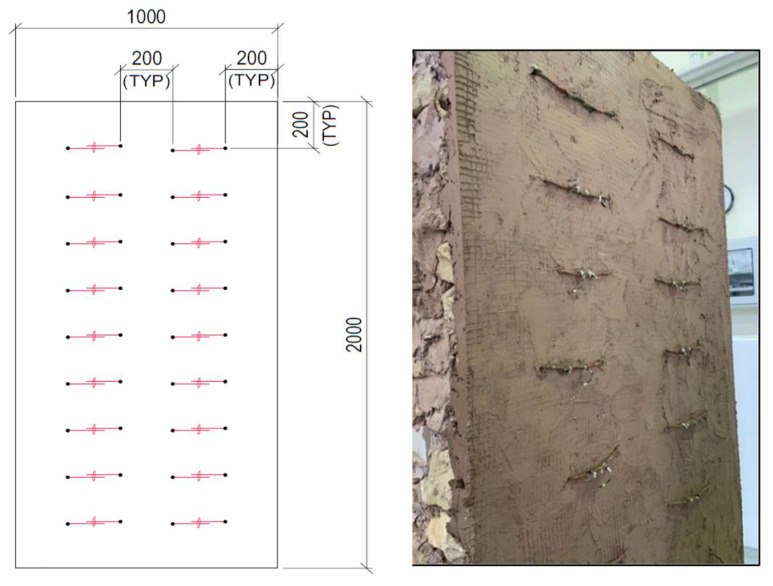
Strengthening of the third wall with one textile layer + ropes (RTR).

The fourth wall was strengthened using a basalt mesh tied with basalt ropes. Holes for the ropes were first drilled into the walls. The distance between the holes was 20 cm in both directions. The ropes were inserted inside the wall and grouted with a high strength epoxy to fill the holes. A layer of sarooj mortar was then applied to the tension face of the wall in preparation for the textile. After applying the basalt mesh, basalt ropes were tied and overlapped horizontally using steel cable ties (see Figure 15). Finally, a second plaster layer of 0.5 cm thick was applied to cover the mesh and left to cure.

## 4. Test Set up for Wall Bending Tests

**Figure 16:**
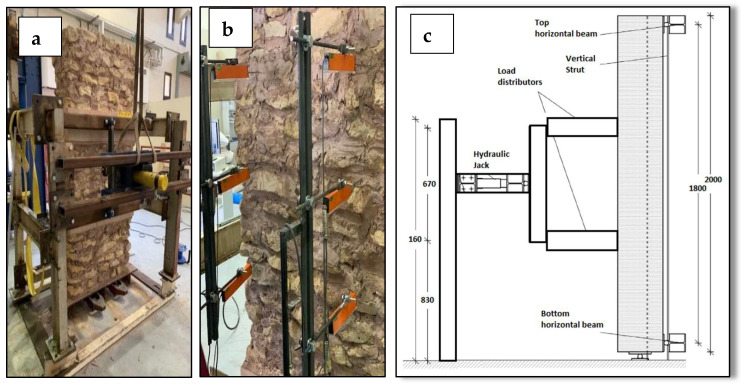
Experimental apparatus for bending tests: (**a**) global view, (**b**) LVDTs, and (**c**) vertical section.

All walls were tested for bending using four-point bending tests. The bending tests were performed by applying two forces laterally against the wall at the top and bottom third of the wall height. A reaction frame was designed and built for this research program, as illustrated in Figure 16. To measure the deflections, displacement transducers were placed on the tension side of the wall face at the top, bottom third, and mid-point (see Figure 16). Application of the force was load controlled until the first cracking; it was then switched to displacement control to prevent overturning and wall collapse.

## 5. Bending Test Results of Stone Masonry Walls

**Figure 17:**
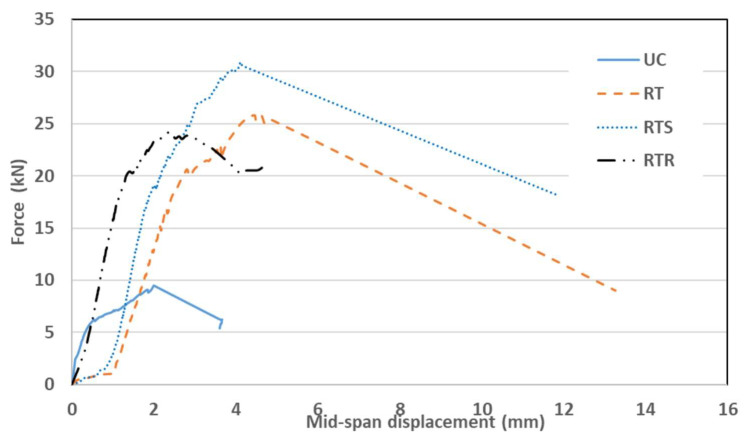
Load vs. midspan displacement for all walls.

**Figure 18:**
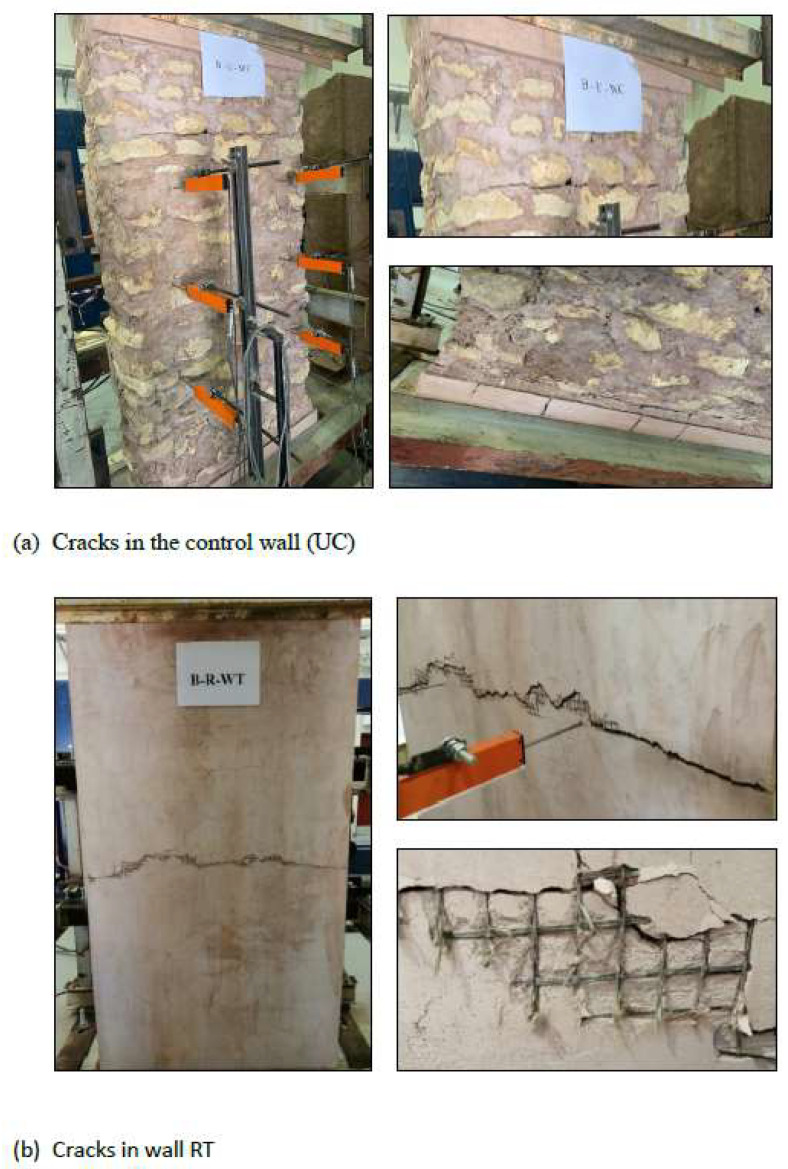

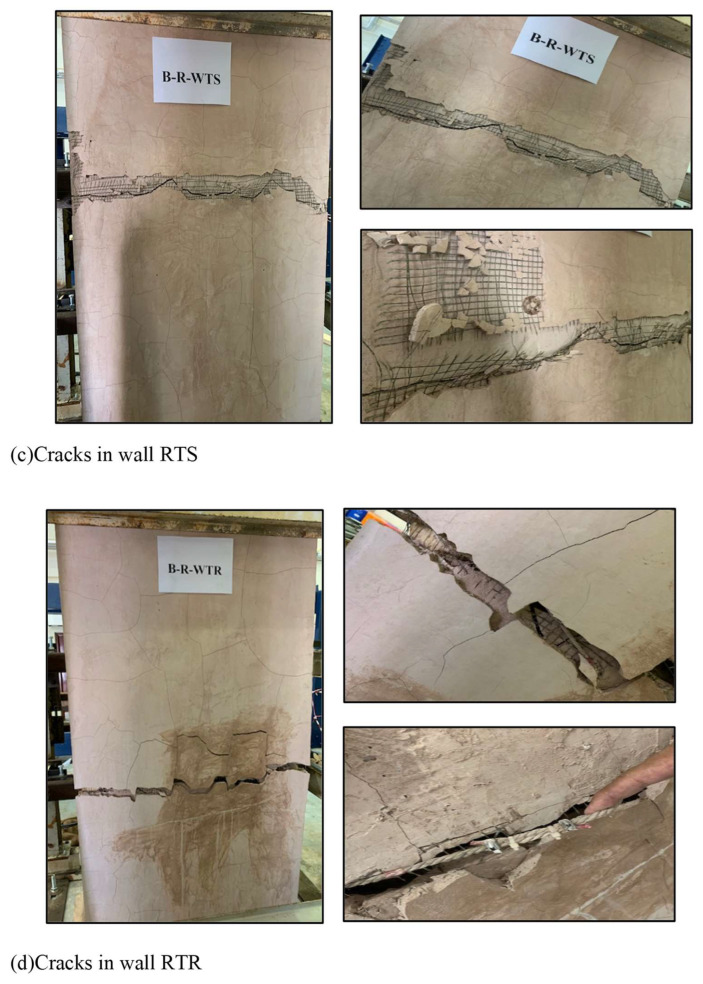
Failure modes and crack patterns.

**Figure 19:**
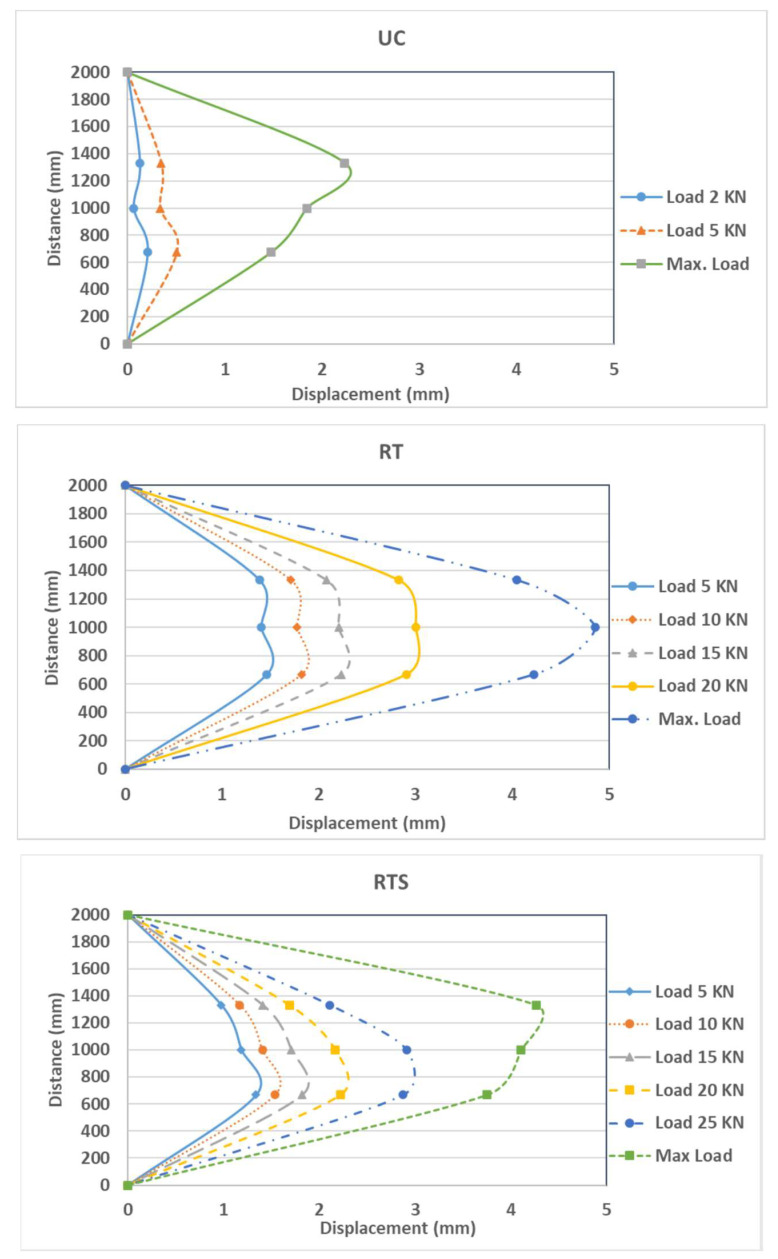

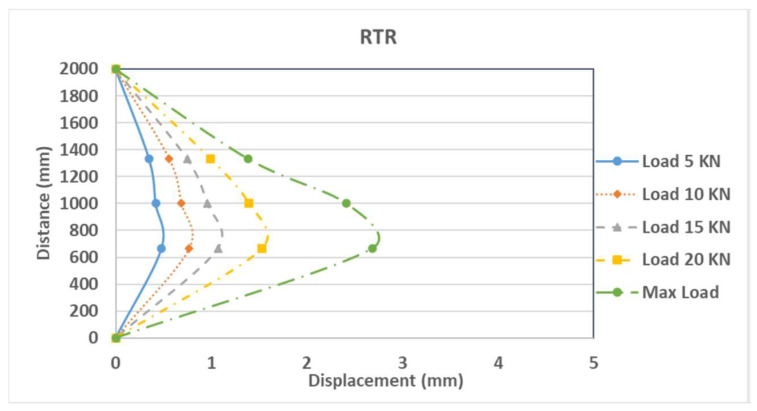
Displacement profile along the wall height.

The load versus out-of-plane mid-span deflections for all wall specimens is shown in Figure 17. For the control wall (UC), the behavior is linear, and no cracks were observed on the wall up to a 6 kN load, as the specimen remained undamaged. At this stage, the wall started to crack on the tension side, around the center of the top third of the wall, and this was followed by other cracks in the bottom of the wall (see Figure 18a). The first crack opened at the top of the wall, since the compressive weight force of the wall was the smallest at the top and highest at the bottom, where it might counteract (due to the normal compression force of the weight) the tensile stress from bending. The crack affected, for the most part, the masonry mortar interface and involved the whole masonry thickness. The slope of the load versus the mid-span deflection shows a decreased stiffness beyond the cracking load. At a maximum failure load of 9.15 kN, the top one-third point exhibited the largest displacement of 1.93 mm. Figure 19 shows the displacement profile along the wall height. The wall displacements are at a maximum at the top third height of the wall where the cracks were first observed.

The wall strengthened with one layer of textile (RT) shows, at the initial stage, a relatively linear behavior and no cracks were observed on the wall up to a 20 kN load. Beyond this load, a horizontal crack started to develop in the mortar at about the mid-height point of the tension side of the wall (see Figure 18b). Consequently, basalt textiles started to bear tension loads, leading to a continuous widening of the crack until failure. The walls failed after the rupture of the textile layer. The crack affected the masonry mortar interface and involved the whole masonry thickness. At the maximum failure load of 25.8 kN, the mid-point exhibited a displacement of 4.61 mm. Figure 19 shows the displacement profile along the wall height. The wall displacement was at a maximum at the mid-height of the wall. The test was stopped after reaching the maximum load to prevent wall overturning collapse. The main crack (where the textile fibers ruptured) involved the whole masonry thickness, and no debonding was observed between the mesh and substrate.

The second strengthened wall (RTS) was strengthened with one layer of textile and augmented with mechanical connecters consisting of brass screws and washers. A similar response of linear behavior was observed before cracking at 18 kN, at which cracks developed around the center of the top third of the wall. A small shift in the load versus the mid-span deflection curve was observed due to the initiation of the crack, with a minor change in the slope of the curve indicating a minor stiffness decrease (Figure 17). This wall performed the best in terms of the maximum load attained compared to the reference wall. At the maximum failure load of 30.9 kN, the mid-point exhibited a displacement of 4.1 mm. Figure 19 shows the displacement profile along the wall height. The wall displacement was at a maximum at the top third of the wall. The effect of the screws’ mechanical anchoring and the stabilizing of the textile mesh obviously augmented the bond strength provided by the mortar. The disadvantage was the extra work required to install the screws. The main crack (where the textile fibers ruptured) involved the whole masonry thickness, and there was no debonding observed between the mesh, substrate, and screws, as shown in Figure 18c.

**Figure 20:**
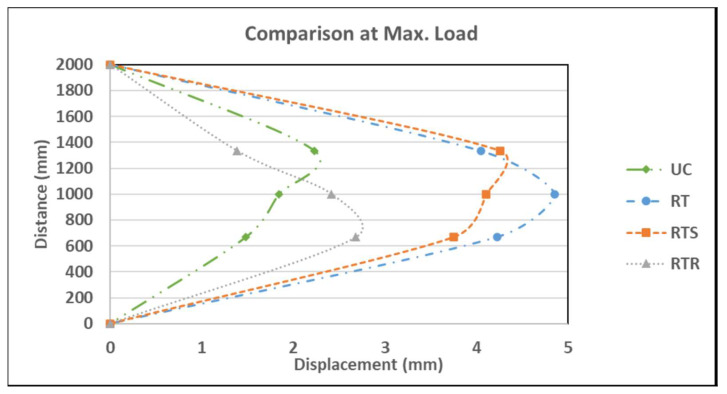
Displacement profile along the wall height at maximum load for all walls.

The last strengthened wall (RTR) was strengthened with one layer of textile augmented with 5 mm basalt rope tied around the textile layer in a horizontal direction at 200 mm intervals. The load versus mid-span deflection is shown in Figure 17. Similar behavior was observed to the other strengthened walls, i.e., a relatively linear behavior until the first crack appeared at a load of 20 kN, corresponding to a mid-span deflection of 1.48 mm. At the maximum failure load of 24.2 kN, the bottom one-third point exhibited the largest displacement of 2.42 mm. Figure 19 shows the displacement profile for the wall height. Using ropes was a laborious job and turned out to be the least effective among the strengthened walls with respect to the maximum resisting load as well as deformation. At the end of the test, it was observed that the tying ropes were loose and relaxed (see Figure 18d), which seems ineffective. The plaster layer might have been disturbed while tying the ropes and this might have caused local pockets of loose mortar layers around the rope area, which in turn affected the overall textile layer. As evidenced, this wall even performed worse than the wall strengthened with textile only (see Figure 20).

The test results are summarized in Table 3. They show that the gain in moment capacity increased (compared to the control wall UC) by 2.78, 3.33, and 2.6 times (or 178%, 233%, and 160%) for the RT, RTS, and RTR walls, respectively.

## 6. Estimating the Moment Capacity of the Strengthened Walls

The theoretical moment capacity of masonry walls retrofitted by TRM can be estimated using analytical models from the available literature [29,30,31]. For the unreinforced control specimen, Equation (1) can be used to calculate the failure moment capacity:

(1) $$M_{u}=\frac{f_{t}bh^{2}}{6}$$

where *f*_t_ is the tensile strength of mortar and *b* and *h* are the width and thickness of the wall, respectively.

**Figure 21:**
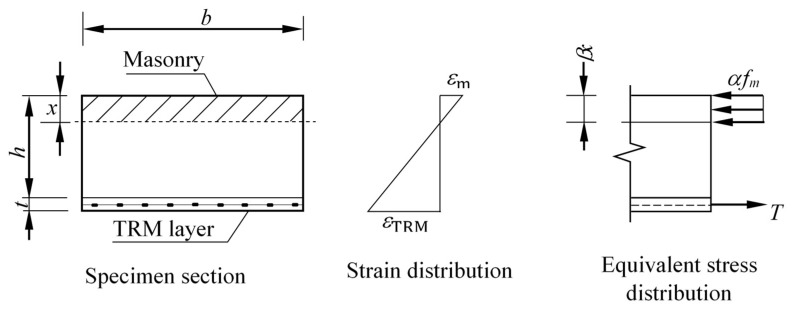
Theoretical ultimate state stresses at flexural failure of the strengthened specimens.

For strengthened wall specimens with failure due to textile rupture, which is the case in this study, the flexural model proposed in the literature is based on the assumptions that the plane section remains plane after bending, the tensile strength of masonry can be neglected, and the equilibrium condition of section forces is satisfied [30]. As shown in Figure 21, the moment capacity can be estimated as follows:

(2) $$T=\alpha f_{m\;}b\;\beta x$$

(3) $$M_{UR}=T\left(h+\frac{1}{2}t-\frac{1}{2}\beta x\right)$$

where *t*, *b*, and *x* are the thickness of the TRM composite layer, the width of the cross-section, and the compressive depth of masonry, respectively. *f*_m_ is the compressive strength of masonry, and *α* and *β* are the equivalent rectangular stress coefficient and stress block depth coefficient, respectively. The values of *α* and *β* were taken as 0.67 and 0.75, as recommended by Deng et al. [31]. The value of *T* was determined from the composite coupons presented earlier. The theoretical values of moment capacity for the strengthened wall are listed in Table 3. Equation (3) reasonably estimates the moment capacity for the wall with screws (RTS) and slightly overestimates the other two walls. This is due to the heterogeneous nature of the stone masonry, which has more variations compared to brick masonry. However, this model may be used to provide a rough estimate of the moment capacity due to its simplicity.

## 7. Conclusions

This research investigated the use of the TRM technique to strengthen historical stone masonry structures. Chemical and physical tests were performed to characterize the materials used in the study: limestone, sarooj, and basalt textile. Then, TRM was used to strengthen masonry walls for out-of-plane bending. One masonry wall was unreinforced, and the rest were reinforced with textile fibers. On the basis of the presented experimental work, the following conclusions are made:

- Sarooj mortar is compatible with stone masonry structures based on the investigated physical and chemical properties of the stones and sarooj. The compatibility of the strengthening system is vital in preserving historical structures.
- The unreinforced masonry specimen (UC) showed sudden brittle failure due to the opening of a single horizontal crack.
- All reinforced specimens exhibited a relatively ductile behavior before failure compared to the URM wall, with increases in the maximum deflection before failure of 130%, 106%, and 21% for RT, RTS, and RTR walls, respectively.
- All strengthened specimens failed due to textile rupture when the basalt textile reached its ultimate strain.
- The strengthened specimens resisted an out-of-plane bending moments of about 2.5 to 3 times that of the unreinforced specimen (a 160–233% increase). The strengthening method using textile mesh in addition to screws and washers (RTS) was the most efficient method.

## Figures and Tables

**Table 1:** Chemical compounds of limestone and sarooj.

Compound	Limestone (%)	Sarooj (%)
SiO_2_	10.33	29.08
TiO_2_	0.73	0.41
Al_2_O_3_	1.27	10.91
Fe_2_O_3_	1.47	11.99
MnO	0.03	0.143
MgO	1.64	11.55
CaO	68.55	24.47
Na_2_O	0.74	4.44
K_2_O	0.1	0.94
P_2_O_5_	0.02	0.15

**Table 2:** Wall identification.

Specimens	1	2	3	4
*Tag.*	UC	RT	RTS	RTR
*Textile*	_	Basalt	Basalt	Basalt
*Specimens*	Control	Strengthened with Textile	Textile + Screws	Textile + Basalt Rope

**Table 3:** Summary of wall bending test results.

Specimen	P_cr_ (kN)	P_u_ (kN)	Δ_cr_ (mm)	Δ_u_ (mm)	M_cr_ (kN.m)	M_u_ (kN.m)	M_u_/M_UC_	M_theor_ (kN.m)
UC	9.51	9.51	0.5	1.99	2.60	2.60	1.0	-
RT	20.21	25.81	2.85	4.61	5.63	7.22	2.78	8.4
RTS	19.11	30.92	1.99	4.11	5.32	8.66	3.33	8.4
RTR	20.25	24.17	1.48	2.42	5.64	6.75	2.60	8.4

Cr = cracking. U = ultimate.

## Data Availability

Not applicable.
